# Guideline-concordant cancer screening among Deaf, DeafBlind, and Hard of Hearing (DDBHH) Puerto Ricans who use American Sign Language

**DOI:** 10.3389/fonc.2026.1771970

**Published:** 2026-02-09

**Authors:** David T. Bruno, Juan J. Fumero-Carrión, Erika Bergeron, Sowmya R. Rao, Francisco J. Muñoz-Torres, Roxana Soto-Abreu, Vivian Colón López, Poorna Kushalnagar

**Affiliations:** 1 Center of Deaf Health Excellence, Gallaudet University, Washington, DC, United States; 2 Department of Global Health, Boston University School of Public Health, Boston, MA, United States; 3 University of Puerto Rico Comprehensive Cancer Center, San Juan, Puerto Rico

**Keywords:** deaf, guideline concordance, health disparities, Hispanic/Latino, Puerto Rico, sign language

## Abstract

**Introduction:**

Puerto Ricans who are Deaf, DeafBlind, and Hard of Hearing (DDBHH) and use sign language are excluded from cancer screening surveillance systems that are conducted primarily in English or Spanish. This exclusion resulted in limited data on screening behaviors among this population.

**Methods:**

We administered a brief National Cancer Institute’s Health Information National Trends Survey (HINTS) to age-eligible Puerto Rican adults through deaf Puerto Rican team members. This survey included items that asked about cancer screenings in American Sign Language (ASL) and English.

**Results:**

Screening adherence was 69% for cervical cancer, 80% for breast cancer, and 64% for colorectal cancer. These rates exceed the colorectal cancer screening rate reported for the general Puerto Rican population (55%).

**Discussion:**

This first assessment of cancer screening behaviors among Deaf, DeafBlind, and Hard of Hearing Puerto Rican sign language users demonstrates that accessible research methods can successfully reach some of excluded populations and documents screening adherence within this population for the first time, while highlighting critical needs to implement evidence-based sign language-fluent health navigation services in Puerto Rico to achieve screening targets and reach Puerto Rican Sign Language-dominant users.

## 1 Introduction

Cancer screening has shown the successful prevention of deaths (1). The United States has established Healthy People 2030 targets for breast, cervical, prostate, lung, and colorectal cancer screening among eligible adults (2). Cancer screening rates among Hispanic/Latino populations vary by cancer type and show disparities compared to national averages. In 2021, Hispanic individuals had lower cancer screening rates than non-Hispanic White individuals for cervical (69% vs. 80%), breast (60% vs. 65%), and colorectal cancer screening (52% vs. 61%) (3). Within this group, Puerto Ricans face particularly complex challenges shaped by migration patterns between the mainland U.S. and the island, further exacerbated by natural disasters, such as hurricanes, and other socioeconomic hardships, which create disruptions in continuity of care and varying healthcare system navigation experiences (4). In addition to reported individual barriers, structural barriers, including geographic accessibility, medical insurance coverage, utilization and knowledge of available benefits for cancer early detection, and provider availability, directly impact screening utilization rates for this population.

When exploring difference within Puerto Rican subgroups, marked barriers that influence guideline-concordant cancer screening are exacerbated when studying individuals who use sign language. Deaf, DeafBlind, and Hard of Hearing (henceforth labeled as DDBHH) Puerto Rican signers may experience additional barriers, including limited access to trained interpreters during medical appointments and accessible materials in sign language. When patient-provider communication is inadequate and there are no trained health navigators fluent in the patient’s language, deprivation of health information can occur. These barriers may be compounded by Adverse Childhood Communication Experiences (ACCEs), one of multiple social determinants of health affecting outcomes among DDBHH people. ACCEs represent forms of early life toxic stress including language deprivation (lack of or reduced early access to language) and communication neglect (ongoing exclusion from family incidental communication) that can impact lifelong health outcomes. Severe deprivation of language since childhood has been associated with increased risks for multiple chronic health conditions, including cancer (5). Puerto Ricans who experienced language deprivation may face increased risks of health information deprivation related to cancer knowledge, compounding disadvantages in accessing and understanding screening recommendations (6, 7).

Current national data reveal significant gaps in understanding cancer screening prevalence among DDBHH Puerto Ricans who use sign language, as existing surveillance systems systematically exclude this population from data collection. While recent analysis of 2022 Behavioral Risk Factor Surveillance System (BRFSS) data from Puerto Rico reported an approximately 11% lower prevalence of colorectal cancer screening among respondents reporting hearing difficulty compared with respondents without hearing difficulty, these estimates are derived from telephone-based surveys conducted in English or Spanish and therefore likely reflect only individuals able to participate in spoken-language interviews, effectively excluding DDBHH Puerto Rican Sign Language (PRSL) users from the sample (8).

Addressing these screening disparities requires accessible educational resources and improved healthcare provider training, as well as fundamental changes to health surveillance methodology to capture data from DDBHH sign language users. ASL-adapted survey methodologies have demonstrated the feasibility of collecting guideline-concordant cancer screening data directly from ASL users, offering an approach to addressing persistent gaps in cancer surveillance for this population (9). As Puerto Rico continues to face difficulties with environmental, economic and healthcare infrastructure, the systematic exclusion of DDBHH sign language users from cancer screening data represents a critical barrier to understanding and addressing their needs. The linguistic landscape adds another layer of complexity: prior scholarship documents the historical use of PRSL alongside the increasing dominance of ASL, particularly through educational institutions, with variation across communities on the island (9). Given this linguistic shift, the current study focuses on ASL-using Puerto Ricans, acknowledging this as both a strength for measurement consistency and a limitation in fully representing PRSL-dominant individuals. This paper addresses the surveillance gap by using primary data gathered from DDBHH Puerto Rican signers through the Center of Deaf Health Excellence’s validated cancer screening measures in ASL, and describes the study methodology, logistics, and lessons learned for future research and implementation efforts in Puerto Rico.

## 2 Methods

### 2.1 Study design and procedures

Following approval from the Institutional Review Board, we conducted a survey using an ASL version of the National Cancer Institute’s (NCI) Health Information National Trends Survey (HINTS) (10) The study, conducted between July 2023 and June 2025, recruited over 1,000 eligible participants from across the United States, including Hawaii and Puerto Rico through invite-back survey using Center of Deaf Health Excellence’s database, local community events, national events, and events held within study catchment area that were organized in collaboration with community partners. Eligible survey respondents were individuals who: 1) self-identified as Deaf, DeafBlind, or Hard of Hearing (DDBHH); 2) were able to communicate in ASL; 3) were age-eligible for screenings; and 4) were residents of the United States or Puerto Rico. For the purpose of this study, residency in Puerto Rico was an inclusion criterion, whereas ethnic self-identification was collected as a demographic variable. The abridged HINTS (HINTS-ASL) survey and required 15 minutes to complete.

### 2.2 Data collection

Data collection was conducted by DDBHH Puerto Rican team members with strong community ties with Puerto Rico, including a Puerto Rico-born individual fluent in both ASL and PRSL. Participants were required to demonstrate comprehension of ASL during the consent process. The survey was administered in ASL to maintain consistency with the validated instrument, and team members could clarify concepts as needed. Participants received a $10 valued cash dollars as a token of appreciation for their participation. Identifying information was kept confidential and separated from survey responses, connected only by a unique identifier.

### 2.3 Measures

Survey items assessed demographics, cancer screening history, and health insurance status, focusing on items aligned with USPSTF cancer screening guidelines [see Kushalnagar et al, 2017 for methodology (10)]. The specific screening questions included:

#### 2.3.1 Colorectal cancer screening

Participants were asked: “*Have you ever taken a colorectal cancer screening test?*” Respondents answering “yes” were asked when their most recent test occurred and which type they completed. Response options included: colonoscopy, fecal occult blood test (FOBT), fecal immunochemical test (FIT), flexible sigmoidoscopy, virtual colonoscopy, and DNA stool test. Guideline-concordant colorectal cancer screening was defined as completing at least one recommended screening test within guideline intervals: colonoscopy every 10 years, FOBT/FIT annually, sigmoidoscopy/virtual colonoscopy every 5 years, or DNA stool testing every 3 years.

#### 2.3.2 Breast cancer screening

Participants were asked: “*When did you have your most recent mammogram to check for breast cancer, if ever?*” Response options included: A year ago or less; More than 1, up to 2 years ago; More than 2, up to 3 years ago; More than 3, up to 5 years ago; More than 5 years ago; I have never had a mammogram. Guideline-concordant breast cancer screening was defined as having completed a mammogram within the past two years.

#### 2.3.3 Cervical cancer screening

Participants were asked: “*How long ago did you have your most recent Pap test to check for cervical cancer?*” Response options included: A year ago or less; More than 1, up to 2 years ago; More than 2, up to 3 years ago; More than 3, up to 5 years ago; More than 5 years ago; I have never had a Pap test. Guideline-concordant cervical cancer screening was defined as having completed a Pap test within the past three years.

#### 2.3.4 Sociodemographic and health indicators

- Functional hearing ability was assessed with the question: “*If a person* sp*eaks to you in a quiet room, how much can you understand what the person says?*” Responses were categorized as understand well (“all/mostly all”) or cannot understand well (“some, little, or none”).
- Education was categorized as “having graduated High School or some college” and “College graduate.”
- Race was categorized as White, Hispanic, and Other (Black/African American, Asian, Other).
- Marital Status was dichotomized into Married/Living with partner and Divorced/Widowed/Separated/Never Married.

Descriptive statistics were calculated for all characteristics – overall and by screening status (adherent/non-adherent). Complete cases were summarized using SAS 9.4 (SAS Institute, Cary, NC, USA).

## 3 Results

### 3.1 Sample characteristics

This study included 79 ASL-using Puerto Rican residents with an average age of 58 years (SD = 6.6, range: 42 to 71 years). **Table 1** presents the demographic and health characteristics of the sample. The majority of participants were female (59.5%) and Puerto Rican (89%). Educational attainment was relatively high, with 81% having completed high school or some college education, and 19% holding a college degree. A majority (80.3%) of the study sample reported difficulty understanding spoken language in a quiet room, and 62.2% self-reported not using English well.

**Table 1:** Distribution of sample characteristics by cancer screening status among Puerto Ricans aged 42–71 years.

Characteristics	Overall (N=79)	Type of Cancer Screening					
		Age-eligible PAP test within 3 years		Age-eligible mammogram within 2 years		Age-eligible Colon Test	
		Not Adherent (n=12)	Adherent (n=27)	Not Adherent (n=9)	Adherent (n=37)	Not Adherent (n=28)	Adherent (n=50)
	N^*^ (Col%)	n^*^ (Row%)					
Age in Years							
42-49	10 (12.7)	0 (0.0)	5 (100.0)	1 (25.0)	3 (75.0)	6 (66.7)	3 (33.3)
50-64	57 (72.2)	12 (36.4)	21 (63.6)	7 (21.2)	26 (78.8)	18 (31.6)	39 (68.4)
65-71	12 (15.2)	0 (0.0)	1 (100.0)	1 (11.1)	8 (88.9)	4 (33.3)	8 (66.7)
Health Insurance							
No	8 (10.3)	1 (33.3)	2 (66.7)	1 (33.3)	2 (66.7)	4 (50.0)	4 (50.0)
Yes	68 (87.2)	11 (31.4)	24 (68.6)	8 (19.0)	34 (81.0)	24 (35.8)	43 (64.2)
Not sure	2 (2.6)	0 (0.0)	1 (100.0)	0 (0.0)	1 (100.0)	0 (0.0)	2 (100.0)
Education							
HS/Some college	64 (81.0)	9 (28.1)	23 (71.9)	7 (17.9)	32 (82.1)	24 (37.5)	40 (62.5)
College graduate	15 (19.0)	3 (42.9)	4 (57.1)	2 (28.6)	5 (71.4)	4 (28.6)	10 (71.4)
Functional Hearing							
Understood all or most of what was said	15 (19.7)	1 (12.5)	7 (87.5)	1 (10.0)	9 (90.0)	5 (35.7)	9 (64.3)
Understood some or none of what was said	61 (80.3)	11 (35.5)	20 (64.5)	8 (23.5)	26 (76.5)	21 (34.4)	40 (65.6)
Use English							
Very well or well	30 (38.0)	2 (15.4)	11 (84.6)	1 (7.1)	13 (92.9)	9 (31.0)	20 (69.0)
Not well or none at all	49 (62.0)	10 (38.5)	16 (61.5)	8 (25.0)	24 (75.0)	19 (38.8)	30 (61.2)
Birth Sex							
Male	32 (40.5)	-	-	-	-	13 (40.6)	19 (59.4)
Female	47 (59.5)	12 (30.8)	27 (69.2)	9 (19.6)	37 (80.4)	15 (32.6)	31 (67.4)

^*^Might not add up to the total due to missing values; Col%=Column Percentage; Row%=Row Percentage.

About 87.2% of the sample had health insurance, while 10% self-reported an additional disability beyond early deafness.

### 3.2 Guideline-concordant cancer screening

Among 39 age-eligible women for cervical cancer screening, 69.2% self-reported being up to date with guideline-concordant Pap testing within the past three years. Descriptive data showed that among the small subgroup who reported understanding all or most spoken language (n=8), 87.5% were guideline-concordant, compared to 64.5% among those who understood some to none (n=31). Similarly, 84.6% of those who used English well (n=13) were guideline-concordant versus 61.5% of those who did not use English well (n=26).

Among 46 age-eligible women in the sample, 80.4% had received a mammogram within the past two years. Within this group, 90% of those who understood all or most spoken language (n=10) were guideline-concordant compared to 76.5% of those with greater difficulty (n=34). A similar pattern was observed for English use, with 92.9% guideline-concordant among those using English well (n=14) versus 75% among those who did not (n=32). Among 78 age-eligible participants, 64.1% self-reported having completed colorectal cancer screening consistent with the recommended guidelines. Unlike cervical and breast cancer screening, guideline-concordant colorectal screening rates were similar regardless of functional hearing ability (approximately 64.3-65.6% across groups). However, colorectal screening adherence rate was slightly higher for individuals who self-reported using English well (69%) compared to those who do not use English well (61.2%).

## 4 Discussion

This ASL-accessible cancer screening survey is the first to assess guideline-concordant cancer screening among DDBHH Puerto Rican adults who were able to complete the survey in ASL. Highlighting these efforts is important to contextualize the inclusivity and linguistic integrity of the sample, which strengthens the validity and interpretation of the data analyses.

Guideline-concordant cancer screening for cervical, breast, and colorectal cancer was comparable to or slightly higher than estimates reported in other non-Puerto Rican samples that also use ASL. Guideline-concordant cervical cancer screening (69.2%) observed in the Puerto Rican sample was similar to the reported 67% guideline-concordant cervical cancer screening among 475 Americans who live outside of Puerto Rico (11). The unadjusted guideline-concordant breast cancer screening rate (80%) is also close to the unadjusted 77% reported in a non-Puerto Rican American sample who answered cancer screening questions presented in ASL and English between 2015 and 2023 (12). Guideline-concordant colorectal cancer screening (64.1%) was higher than the 52% reported in a sample of 600 ASL-using adults who completed colorectal screening questions presented in ASL and English (13).

While descriptive data suggested numerically higher screening rates among those with better functional hearing and English proficiency for cervical and breast cancer, these findings were based on very small subgroups (8–13 individuals) and require cautious interpretation. DDBHH women who use primarily sign language to communicate on a daily basis are at disadvantage for accessing preventive screenings, and could benefit from sign language-fluent navigator service. However, Puerto Rico does not have sign language-fluent community health navigation services. Sign language-fluent community health navigation involves language-concordant professionals who communicate directly in the patient’s primary language and provide navigation support throughout the screening process, and this model has been identified as a core strategy for improving cancer screening access among ASL-fluent adults (14). Despite the gap of sign language-fluent health navigators in Puerto Rico, existing cancer control infrastructure presents opportunities for improvement. Puerto Rico’s Comprehensive Cancer Control Program provides a framework that could support language concordance through development of plain language educational materials, workforce training to improve patient-provider communication, and regional cancer education initiatives aimed at strengthening cancer literacy across the territory (15).

Additionally, the island’s network of Federally Qualified Health Centers (FQHCs), which serve as safety-net providers for underserved populations, could be leveraged to develop targeted screening programs for patients who rely on sign language to access information. These FQHCs have obligations under Title VI of the Civil Rights Act and Section 504 of the Rehabilitation Act (disability rights) to provide access and effective communication for patients with limited English proficiency and disabilities, respectively (16). Together, these requirements and the existing role of FHQCs in cancer screening position them as potential partners for improving accessibility of cancer screening services. Adapting the sign language-fluent navigation model within these existing systems may help improve screening participation, particularly among those who may experience barriers to screenings. FQHC centers already receive federal funding to ensure access for all residents, including health navigation support, which could be leveraged to support systematic health navigation services provided by trained, health-literate individuals who are fluent in ASL and have lived experiences (17).

Our unadjusted findings reveal important insights when contextualized within national health objectives and territorial screening patterns. While the study sample of DDBHH Puerto Rican signers almost reached the Healthy People 2030 target^2^ of 77.1% for breast cancer screening (80.3% up to date), gaps remain for cervical (69.2% vs. 79.2% target) and colorectal cancer screening (64.1% vs. 72.8% target).

Among women eligible for cervical screening, 87.5% who understood all or most of spoken language in a quiet room were guideline-concordant versus 64.5% who understood some or none of what was said in a quiet room. Similar patterns were observed for mammography (90% vs 76.5%). However, these descriptive differences are based on small subgroups and were not statistically tested. Only 55.5% of the general Puerto Rican population aged 45–75 years met colorectal cancer screening recommendations as of 2022, suggesting that our ASL-using sample’s 64.1% adherence rate is performing better compared to their counterparts. These comparisons underscore both the progress made and the continued need for accessible screening interventions among non-English users to achieve national health equity goals.

This study has several strengths. Most notably, it includes sign language-using DDBHH Puerto Rican adults, a group rarely represented in surveillance datasets. The ASL-accessible survey enabled participants to report their screening history in a language they were comfortable using, providing valuable data that are largely absent from the literature. Recruitment was further strengthened by deaf Puerto Ricans with strong community ties who boosted participation at key “hub” sites with established sign language community presence, including the deaf school, sign language-accessible churches, and regular deaf gatherings at cafés in specific areas of the island.

This study also has limitations. The modest sample size restricts between- and within-group analyses that could further characterize screening behaviors and factors influencing guideline-concordant cancer screening among DDBHH Puerto Ricans who use sign language. The relatively small, community-recruited sample also limits the extent to which the reported findings can be generalized and may not fully represent the broader population of deaf adults who use ASL and live in Puerto Rico, particularly those who are less connected to community organizations or who differ in socioeconomic or regional characteristics. The small number of participants reporting good functional hearing or English proficiency limits our ability to draw meaningful conclusions about these characteristics. Also, the small cell sizes limit the ability to adjust for other potentially confounding founders that may influence screening behaviors. Larger, adequately powered studies are needed to confirm whether functional hearing and language proficiency meaningfully affect screening uptake in this population. Additionally, some potential participants were unable to attend recruitment events because of transportation barriers or limited access to information. The use of a validated ASL survey instrument strengthened measurement consistency; however, it also meant that PRSL-dominant signers, who are more often found outside major urban centers, were less likely to be included. Future research should therefore prioritize adapting cancer screening measures into PRSL and partnering with PRSL-using communities to capture the full spectrum of linguistic experiences of deaf Puerto Ricans. Pooled analyses should be avoided so to better distinguish individuals who use English well from those who do not.

## Data Availability

The datasets presented in this article are not readily available because the participant is from a hard-to-reach population that is small and risks being identified. Requests to access the datasets should be directed to poorna.kushalnagar@gallaudet.edu.
